# On-site breakfast provision in early childhood education and care (ECEC) services in Australia: a multi-method investigation

**DOI:** 10.1007/s00394-025-03590-4

**Published:** 2025-02-01

**Authors:** Seon Y. Park, Miaobing Zheng, Kathleen E. Lacy, Karen J. Campbell, Penelope Love

**Affiliations:** Institute for Physical Activity and Nutrition (IPAN), School of Exercise and Nutrition Sciences (SENS), Deakin University, Geelong Waurn Ponds Campus, 75 Pigdons Road, Geelong, VIC 3216 Australia

**Keywords:** ECEC, Early childhood education services, Onsite breakfast provision quality, Enablers and barriers of healthy breakfast provision, childcare

## Abstract

**Background:**

Breakfast is vital for young children’s health. In Australia, breakfast is often provided in government-approved Early Childhood Education and Care (ECEC) services. However, research on breakfast provision in Australian ECEC services is limited. This study aimed to evaluate breakfast quality and enablers and barriers of breakfast provision in these settings.

**Methods:**

A multi-method, sequential explanatory design was employed, including survey, on-site visits, and semi-structured interviews. ECEC services offering breakfast in Victoria, Australia, participated in an online survey to assess breakfast provision quality based on Victorian Health Eating Advisory Service (HEAS) guidelines. Multivariate logistic regression was utilised to identify the relationship between breakfast quality and service characteristics. A subset of services participated in 1-day observational visits and 30-min semi-structured interviews. The on-site visits observed breakfast foods and environments, while interviews explored enablers or barriers of breakfast provision.

**Results:**

Fifty ECEC services participated in the online survey, with common breakfast items such as cereal, bread, and milk being frequently provided, while fruits and vegetables were among the least common items offered. Only 10–16% of services met the HEAS definitions of high-quality standards, and these centres were mostly located in socioeconomically advantaged areas. Of these, four services participated in the on-site observation phase, where the use of full-cream milk and multigrain bread was commonly noted. Additionally, eight interviews (two from each centre) were conducted to explore enablers and barriers to healthy breakfast provision. Key enablers included government funding and the use of nutritional guidelines, while barriers involved time constraints, budget limitations, staffing shortages, and insufficient confidence in applying and utilising nutrition guidance.

**Conclusion:**

Fruits and vegetables are rarely provided at breakfast in ECEC settings, and only a small number of ECEC services met high-quality breakfast standards, with those in socio-economically advantaged areas more likely to achieve these standards. Targeted interventions, particularly in disadvantaged areas, are essential to improve the quality of breakfast provision, with a focus on including fruits and vegetables in line with guidelines. While the findings of this study had jurisdictional limitations, this study highlighted the importance of addressing issues such as time constraints, budget limitations, and staffing shortages, along with establishing practical and clear breakfast guidelines to enhance the quality of breakfast provision in ECEC settings. Further research is needed to explore specific and actionable strategies for implementing these improvements.

**Supplementary Information:**

The online version contains supplementary material available at 10.1007/s00394-025-03590-4.

## Introduction

Breakfast is often regarded as the most important meal of the day, particularly vital for young children under 5 years of age [1]. Consuming a well-balanced breakfast equips children with the energy and stamina required for the physical demands of playing [2], learning [3], and growing [4]. In particular, breakfast plays a crucial role in brain development and function [3]. Nutrients consumed during breakfast, such as iron, B vitamins, and protein, are known to improve concentration, problem-solving abilities, and memory [3]. For this reason, a child’s attention span, mood, and even the capacity to comprehend complex tasks can be enhanced by ensuring a consistent and nutrient-dense first meal of the day [3]. Moreover, regular breakfast consumption is intricately linked with the intake of essential vitamins and minerals, which are fundamental to a child's overall health and developmental trajectory [4]. For example, nutrients like calcium, fibre, and essential fats, often found in typical breakfast foods (e.g., breakfast cereals, milk, and yoghurt), are crucial for bone development, digestive health, and brain function, respectively [5]. As the early childhood years are characterised by swift growth and numerous developmental milestones, a lack of essential nutrients can lead to nutrient deficiencies, associated health complications, and may have broader implications for overall development [6]. Regular breakfast skipping can also instil poor eating habits that may persist into adulthood, potentially leading to long-term health concerns [7].

Australian national surveys have reported that breakfast consumption is common among a majority of children. One study reported that 80% of children aged 2–17 years consumed breakfast [8], with 94.8% of children aged 2–3 years and 92.2% of 4–5 years regularly consuming breakfast. Another found that 90% of children aged 2–16 years were regular breakfast consumers [9]. While these national data generally indicated a high rate of breakfast consumption among young Australian children, these results were often grouped for children aged 2–3 years and 4–5 years or aggregated for the entire age range of 2–5 years. To date, there hasn't been a breakdown of breakfast consumption specific to each year of age for Australian children under 5 years.

Also, while several studies have identified the proportion of breakfast consumers or skippers among young children [8–11], there is limited research focusing on the nutritional quality of breakfasts among children. A study by Park et al. revealed that the quality of breakfast among young Australian children decreased from 4.8 to 2.7 points out of a total of 5 points as they aged from 1.5 to 5.0 years [12]. However, it is important to further explore how the location where breakfast is consumed may impact the nutritional quality of breakfast children eat.

In Australia, ‘ECEC’ is referred to as Early Childhood Education and Care (ECEC) [13]. The most common forms of ECEC accessed by Australian children are Centre-Based Day Care services, such as 'Kindergarten,' 'Long Day Care (LDC),' or a combination of both, where kindergarten programs are integrated into LDC settings [13, 14]. 'Kindergarten' is often referred to as 'kindy' in certain jurisdictions and is designed for children aged 3 to 5 years, serving as a formal educational foundation before they transition to primary school. LDC provides extended care (at least 8 h/day) for children under 5 years of age and is often accompanied by on-site food services [14]. There has been a notable increase in the enrolment of ECEC services in recent years. In 2022, 48.2% of Australian children aged 0–5 years (n = 1,335,660) were enrolled in approved ECEC services, reflecting a 1.4% increase (n = 18,650) compared to 2021 [13]. Of these, 851,210 children (59.7%) were in Centre-Based Day Care services, spending an average of 27.3 h per week in care [14].

Given the increasing popularity and extended hours of operation, ECEC services are strategically positioned to significantly influence the dietary habits of young Australian children [15]. Within these settings, it is recommended that the food served should cover approximately 50–60% of the child’s daily nutritional requirements [15]. Therefore, ECEC services should be considered an emerging setting to monitor the quality of food provided due to the shared aspect of food provision between parents/guardians and ECEC services. Notably, if children are in care for more than eight hours, extra meals and/or midmeals (breakfast or late afternoon tea (snack)) should be provided [16]. However, much of the existing nutrition research in ECEC concentrates on main meals and snacks (i.e., morning/afternoon tea (snack) and lunch), with very limited data addressing breakfast provision. The most recent data documenting breakfast consumption in ECEC was collected nearly 30 years ago, in 1995 [17]. During that time, only a small fraction of children, comprising 1.4% of boys and 2.6% of girls aged 2–3 years, were reported to have breakfast in ECEC, according to data from the 1995 National Nutrition Survey [17].

With the increasing participation of women in the workforce and the consequent rise in the utilisation of ECEC services [14], it is reasonable to assume that many children now begin their day at ECEC services and may be eating breakfast there. Currently, Australia does not have national ECEC food guidelines, but specific guidelines are available in Victoria to assist ECEC centres in providing meals, including breakfast [15]. In particular, Victoria's Healthy Eating Advisory Service (HEAS) recommends a balanced breakfast containing whole grains, dairy products, fruits and/or vegetables, and cereals with minimal sugar content when providing breakfast on-site in ECEC services [15]. However, these guidelines are not mandatory, and their interpretation and implementation may vary widely across Victorian jurisdictions [18]. Given the voluntary and ad hoc nature of these breakfast guidelines, the degree to which Victorian ECEC centres comply with them remains unknown.

Considering operational opportunities and challenges in a setting to understand the breakfast provision quality, a deeper investigation is needed into how breakfast is provided in ECEC services [19, 20]. There is also a need to explore ECEC staff perceptions concerning the provision of nutritious breakfasts, including enablers and barriers. While prior research has extensively explored the quality of main meal provisions in ECEC settings [21, 22] and perceptions among ECEC staff about these main meals [19], a substantial gap exists regarding the quality and perceptions of breakfast provision. Therefore, the aims of this study are to (1) describe the quality of on-site breakfast provision in relation to Victorian ECEC service characteristics, and (2) explore ECEC staff perceptions regarding breakfast provision, including enablers and barriers to quality breakfast provision in Victoria, Australia.

## Materials and methods

### Study design

**Fig. 1:**
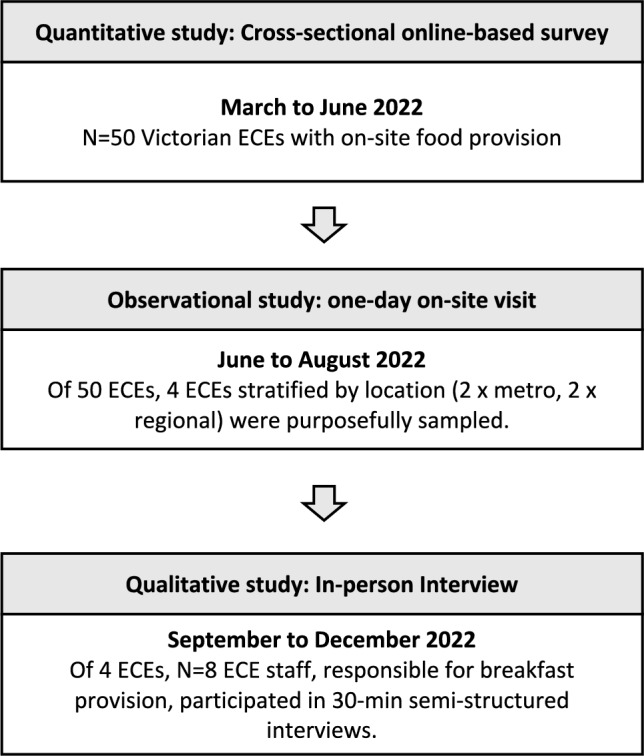
The Multi-method study framework using three phases of study that was conducted from March to December 2022

This study was conducted between March and December 2022 using a multi-method, sequential explanatory design. Quantitative data was collected via cross-sectional online survey (March-June 2022) followed by 2.5 h on one-day onsite observational visits (June–August 2022); and qualitative in-depth staff interviews (September-December 2022) (Fig. 1). This study was approved by the Deakin University Ethics committee [HEAG-H 161_2021].

### Cross-sectional online survey

#### Recruitment and study population

As a pragmatic decision was taken to include this study [23], all Victorian ECEC services listed on the Australian Government’s ECEC website as offering onsite food provision (n = 1068 as at Quarter 1 – from January to March 2022) [24] were emailed information describing the study, with a link to a 10-min online survey. ECEC services were asked to indicate their eligibility in terms of a) operating at least 8 h/day for 48 weeks/annum and b) providing breakfast on-site. ECEC directors were asked to complete the survey. Survey responses were returned directly to the researchers to ensure respondent confidentiality. Respondents received $20 AUD voucher for their survey completion.

#### Data collection and analysis

The revised Environmental Policy Assessment and Observation (EPAO) tool for Australia [25] was used to inform the development of the online survey. Originally developed in the United States to encourage children’s healthy eating and physical activity within ECEC services [26], a previous study made slight adjustments to the EPAO tool from the United States to better suit the assessment of food provision and feeding practices within the context of Australian ECEC [25]. By using the revised EPAO tool for Australia [25], educators in ECEC centres across Australia can effectively identify both strengths (i.e., enablers) and areas requiring improvement (i.e., barriers). In the domain of Nutrition, the tool also assesses the types of foods offered, the methods of food preparation, and overall dietary practices specific to the Australian ECEC context [25].

The survey comprised 20 items, collecting various information on centre characteristics and breakfast provision. The collected data included demographic details about centre management type (for-profit, not-for-profit), geographic location (metropolitan, regional), Socio-Economic Indexes for Areas (SEIFA) level (low, medium, high), overall national quality framework scores (working towards, meeting, exceeding), breakfast provision type (progressive, standard, flexible), and whether a cook/chef is involved in breakfast preparation. It also covered the average daily number of participating children, total number of staff during breakfast, and breakfast timing.

Additionally, the survey recorded the specific breakfast items offered, including items such as cereal, bread, butter, milk, fruits, vegetables, porridges, and beverages, which are typically consumed during breakfast in Australia. Respondents were able to provide details on additional breakfast items, including product type (e.g., white or brown bread) and brand names, through open-ended questions. This comprehensive data is accessible in supplementary file 1.

Survey data on breakfast food items offered were assessed using two Australian guidelines developed by the Victorian government-led Healthy Eating Advisory Service (HEAS) to support the provision of nutritious meals for children, namely (1) Healthy Choices Food and Drink Classification Guide [27]; and (2) the HEAS Breakfast Menu Recommendations for ECEC Services [15].

##### Assessment #1: HEAS healthy choices food and drink classification guide

Using the HEAS Healthy Choices Food and Drink Classification Guide, survey breakfast food item data were scored using the 'traffic light' classification of Green, Amber and Red [27]. Foods/beverages classified as Green are those considered to be from core food groups (e.g. high-fibre breakfast cereal, reduced fat milk, cheese/yoghurt, vegetables, etc.) and of highest quality, with foods/beverages classified as Red (e.g. biscuits, sweet pastries, butter, cream, etc.) being of lowest quality.

According to the guidelines provided by HEAS, quality of breakfast provision was defined as follows for this study:

- High quality: > 50% of Green items and < 20% of Red items
- Medium quality: > 50% of Green items and > 20% Red items
- Low quality: < 50% of Green items and > 20% of Red items

##### Assessment #2: HEAS breakfast menu recommendations for ECEC services

Survey breakfast food data were also assessed based on the five HEAS Breakfast Menu Recommendations [15], namely, that breakfast contains (1) wholemeal/wholegrain choices, (2) milk, yogurt, cheese, or dairy alternatives, (3) cereal products with less than 15g of sugar per 100g if dried fruit is not an ingredient or less than 25g/100g if dried fruit is an ingredient, (4) fruit or (5) vegetables. The score was specifically developed for this study to assess breakfast quality and indicates the number of recommendations met.

- High quality: 5 out of 5
- Medium quality: 3–4 out of 5
- Low quality: < 3 out of 5

Descriptive statistics of ECEC characteristics (i.e., centre characteristics, general characteristics of ECEC staff, breakfast provision type) were conducted. Multivariable logistic regression was used to assess the association between breakfast provision quality and centre characteristics. The exposures were all categorical regarding centre characteristics, including centre management type (for-profit/not-for-profit), location (metro/regional), socio-economic disadvantage (high vs medium/low), overall service level NQS benchmarks (exceeding vs meeting/working towards), and nutrition training (yes/no). The outcome was the high quality of breakfast provision. Stata 16.0 was used for statistical analysis.

### Observational on-site visit and semi-structured interviews

#### Recruitment and study population

On-site visits and follow-up interviews were conducted to supplement the cross-sectional survey data, providing in-depth insights into the specific details of breakfast food items provided. A purposeful subsample of survey respondents, comprising ECEC centres from both regional and metro locations, was selected for on-site visits and follow-up interviews. Centres that provided consent to be contacted were invited to participate. The recruitment process involved contacting centres via email and/or phone twice to confirm their willingness to participate before the week of on-site visits. Consenting staff were contacted for an interview 2–4 weeks after conducting the onsite visit. The on-site observational visits and interviews were conducted by the same researcher (SP). Centres received a $50 AUD gift card voucher for participating in the onsite observation, with interviewees receiving a $20 AUD gift voucher for their time.

#### Observational on-site visit: data collection and analysis

Observational data were collected using a purposely-developed checklist, which was informed by the Environmental Policy Assessment and Observation (EPAO) tool [25]. The EPAO tool comprises two parts: nutritional domains and physical activity domains. Only the nutrition domain was included for the purposes of this study. The checklist was developed by S.P. and further revised by P.L. to accurately reflect the breakfast environment in ECEC services. This evaluation involved capturing specific details such as brand names of breakfast products, nutritional information of breakfast items, the number of children present, and the timing of breakfast provision, etc. (Supplementary file 2). During the designated observation period from 6:30 am to 9:00 am, S.P. conducted observations at four ECEC centres across Victoria, Australia. These observations encompassed the entire breakfast timeframe, from the opening of the centres until breakfast concluded.

#### Semi-structured interviews: data collection and analysis

Results from the on-site visit were used by S.P and P.L. to inform the design of the semi-structured interview guide based on EPAO tools [25], ensuring questions were both comprehensive and relevant to the context. The interview guide (Supplementary file 3) focused on capturing a broad range of staff perceptions, specifically addressing enablers and barriers to on-site breakfast provision within their ECEC centre. All interviews were recorded with consent obtained prior to and verbally reconfirmed at time of interview. All interviews were conducted by S.P. and were approximately 30 min (range 22–36 min) in duration. All interview recordings were transcribed verbatim by S.P., with interview transcriptions reviewed for accuracy by P.L.

All interviews were transcribed, reviewed, and analysed by S.P., using deductive coding based on the interview questions, followed by inductive theming to construct a theoretical framework for content categorisation. This coding process involved analysing interview data to capture and group participants' viewpoints and experiences, particularly those related to enablers and barriers in providing healthy breakfasts at ECEC centres. To support coding framework reliability, P.L. double-checked the coding for, two of the eight interviews. NVivo software (NVivo version 12, QSR International Pty Ltd., Melbourne, Australia) was used to manage the data.

#### Positionality and epistemology in qualitative data analysis

Qualitative data analysis, collected as part of on-site observations and interviews, was underpinned by a contextualist epistemology, emphasizing that knowledge is derived from and is deeply connected to the data's context [28]. The understanding of breakfast provision in LDC centres is a co-constructed reality, emerging from the intertwined experiences and interpretations of the study participants and the researchers (SP, PL, KC, MZ, and KL). This study utilised a combination of quantitative and qualitative data collection methods, particularly interviews, to prioritise the voices and experiences of participants, providing a richer understanding of the context. Regarding positionality, all researchers have nutrition expertise, and some have lived experience with children attending ECEC services. This combined positionality enriched the research process, offering both depth and breadth in understanding the nuances of breakfast provision in ECEC centres. Reflexivity was incorporated throughout the research process, with researchers regularly reflecting on how their positionality and assumptions could influence the data. Their nutrition expertise and lived experiences enriched the analysis, while ongoing critical reflection helped ensure transparency, credibility, and a balanced interpretation of findings.

## Results

### Cross-sectional survey findings

#### Sample characteristics

The characteristics of participating ECEC centres are presented in Table 1. A total of 50 ECEC centres completed the online survey, representing approximately 5% of Victoria’s ECEC centres with onsite food provision (n = 1063; as at Quarter 1—From January to March 2022). Of these, about two-thirds were located in a metropolitan area, with medium or high area-level socio-economic advantage, had a for-profit service management type, had no chefs/cooks involved in breakfast food provision, and were meeting National Quality Standard (NQS) benchmarks. Total median number of children eating breakfast per centre was 19, representing 15.8% of centre enrolments. The mean number of staff available during breakfast was 3.

**Table 1:** Demographic characteristics on Early Care and Education (ECE) services who completed the online survey (n = 50)

	**N (%)**
**Type of service**	
For-profit	38 (76%)
Not-for-profit	12 (24%)
**Geographic location**	
Metropolitan	33 (66%)
Regional	17 (34%)
SEIFA level *	
Low (1–3 decile)	16 (32%)
Middle (4–7 decile)	25(50%)
High (8–10 decile)	9 (18%)
**Overall National Quality Framework scores**	
Working towards	8 (16%)
Meeting	27 (54%)
Exceeding	10 (30%)
**Breakfast provision type**	
Progressive	33 (66%)
Standard	16 (32%)
Flexible	1 (2%)
**Cook/chef involved in breakfast food provision time**	
Yes	18 (36%)
No	32 (64%)
	**Median (minimum; maximum)**
Total number of enrolment children	120.5 (35; 545)
Total number of working staff	30 (5; 60)
Total number of children eating breakfast	19 (1; 50)
Total number of staff during breakfast	3 (1; 8)

^*^Socio-Economic Indexes for Areas (SEIFA) level

#### Frequency of provision of breakfast food items

**Fig. 2:**
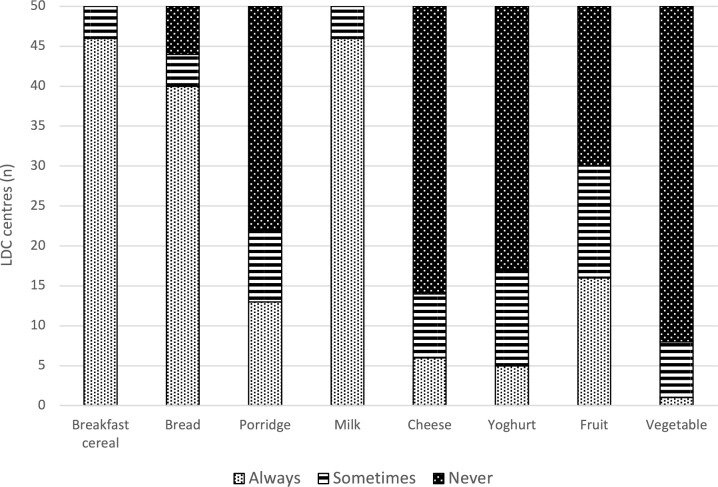
Frequency of provision of breakfast food items based on core food groups (i.e., grains, dairy products, fruit, and vegetable) in ECE services (n = 50)

Breakfast cereal, bread, and milk were more likely to be “Always” offered, with fruits, yogurt, cheese, and porridge more likely to be “Never” or “Sometimes” offered at breakfast. Notably, vegetables were the most likely to be “Never” provided at breakfast (Fig. 2).

#### Quality of breakfast provision

Of 50 centres, the majority of ECEC centres (over 80%) offered medium and low-quality breakfasts, with 16% and 10% meeting the criteria for high-quality breakfasts based on the HEAS ‘Healthy Choices Food and Drink Classification Guide’ (traffic light system) and HEAS ‘Breakfast Menu Recommendations for ECEC Services, respectively (Table 2).

**Table 2:** Breakfast quality for ECE services that participated in the online survey (n = 50) based on HEAS breakfast menu recommendations ^a)^ and healthy choices food and drink classification guide (Traffic light classification) ^b)^

**HEAS Breakfast Menu Recommendations**			**Healthy choices food and drink guide**		
	N	%		N	%
High quality (5/5)	5	10.0	High quality (> 50% green, < 20% amber/red)	8	16.0
Medium quality (3–4/5)	37	74.0	Medium quality (> 50% green, > 20% amber/red)	28	56.0
Low quality (1–2/5)	8	16.0	Low quality (< 50% green, > 20% amber/red)	14	28.0

^a)^ Menu Guidelines for Long Day Care (2020), Healthy Eating Advisory Service (HEAS), Victoria, Australia

^b)^ Healthy Choices Food and Drink Classification Guide (2021), Department of Health, Victoria, Australia

Based on the HEAS guidelines, ECEC centres that offered fruits and vegetables received higher quality scores, and centres that provided muffins, banana bread, or vegemite (a high salt yeast extract spread) received lower quality scores. Overall, ECEC centres in high socio-economic advantaged area had higher odds of offering a high-quality breakfast (OR: 3.04, 95% CI: 1.00, 9.16). However, the quality of breakfast did not significantly differ based on other centre characteristics, including location, centre management type, and nutrition training, as shown in Table 3.

**Table 3:** Multivariable logistic regression between ECE services characteristics with the quality breakfast provision (high vs low) by HEAS Breakfast Menu Recommendations ^a)^ and Healthy Choices Food and Drink Classification Guide (Traffic light classification) ^b)^ (n = 50 sample size)

Service characteristics	HEAS Breakfast Recommendations			Healthy Choices Guide		
	OR	95%CI	P-value	OR	95%CI	P-value
^c)^ Location (Metro vs regional)	1.00	1.00	1.00	4.20	0.01–13.41	0.73
Service management type (For-profit vs not-for-profit)	4.47	0.21–95.6	0.34	0.33	0.01–7.78	0.50
^d)^ Socio-economic advantage level (High vs medium/low)	3.04	1.00–9.16	0.04*	0.27	0.07–1.05	0.06
^e)^ Overall NQS score (Exceeding vs meeting/working towards)	13.28	0.22–48.11	0.22	14.02	0.01–48.6	0.53
Nutrition training (Yes vs no)	0.41	0.02–0.39	0.54	1.51	0.05–50.0	0.82

^a)^ Menu Guidelines for Long Day Care (2020), Healthy Eating Advisory Service (HEAS), Victoria, Australia

^b)^ Healthy Choices Food and Drink Classification Guide (2021), Department of Health, Victoria, Australia

^c)^ Regional area including both inner and outer regional Australia (ABS 2021)

^d)^ High (7–10 SEIFA decile), Medium (4–7 SEIFA decile), Low (1–3 SEIFA decile) (ABS 2022)

^e)^ According to ACECQA National Quality Standard report as at Q1 2022 (The Australian Children’s Education and Care Quality Authority; ACECQA)

### Observational on-site visit findings

Four ECEC centres were purposefully recruited for the observational study, with two located in metropolitan areas and two in regional areas. In terms of management type, three centres were 'for-profit,' while one was 'not-for-profit'. On-site breakfast provision appeared to be consistent across all centres, regardless of location or management type.

During the on-site observations, it was evident that the timing of breakfast provision was consistent among all four ECEC centres. Specifically, three centres offered breakfast between 7 am and 8:30 am, while one centre extended its breakfast service hours from 6:45 am to 8:30 am. This adjustment was made in response to children expressing hunger during these early hours, demonstrating the centres’ adaptability in meeting the needs of the children.

Observations also revealed that each centre typically began breakfast service when around 2–3 children gathered (usually at 7 am) with two educators present. Around 8 am, the number of children having breakfast increased significantly, reaching nearly 15–20 children, irrespective of their age group, all participating in breakfast in the same room. At this time, an additional educator would arrive, resulting in a total of three educators coordinating on-site breakfast provision. One educator was responsible for welcoming and greeting the children as they arrived, another assisted in serving breakfast, and the third managed the cooking and preparation tasks, such as toasting bread and pouring cereal. It appeared that no staff were specifically available to supervise the children who had already finished their breakfast. Children who finished breakfast stayed in the same room, engaging in free play with toys or books until additional educators or children arrived.

In terms of breakfast service style, all observed ECEC centres adopted a progressive meal provision approach. A progressive mealtime is characterised by an extended period during which food is made available to children, and the decision about when and how long to participate in the breakfast time is driven by the children themselves. Three of the ECEC centres offered breakfast cereals such as Weet-Bix ™, Nutri-Grain ™, Rice Bubbles ™, and Cornflakes ™, with one centre providing cooked oats porridge. All observed ECEC centres provided wholegrain or multigrain bread options, with no provision of white bread. Full-fat milk was served exclusively, with no options for reduced-fat milk, skim milk, or plant-based milk alternatives. No fruits or vegetables were included in the breakfast meal options at any of the centres. Also, breakfast provision typically consisted of a simple menu and a small group of children, allowing educators to easily manage and be aware of any specific food allergies. Educators were informed of children's allergies and took appropriate precautions. However, for children with allergies, there was often a lack of alternative breakfast options, and allergy-related foods were simply excluded from their meals rather than replaced with suitable substitutes.

### Qualitative interview findings—enablers and barriers to providing healthy breakfast at ECEC services

A total of eight ECEC staff, responsible for breakfast provision, were recruited for interviews across the four ECEC centres (2 from each centre; M1-M4 and R1-R4) involved in on-site observational visits. Findings from the qualitative analysis and example quotes are presented in Table 4 and described below. Two main enablers were identified – the potential of a government-funded breakfast program and consistent nutrition guidelines. Four main barriers were identified – time constraints, budgetary limitations, limited awareness of nutrition guidelines, and insufficient staffing.

**Table 4:** Enablers and barriers to providing healthy breakfast across Victoria, Australia from semi-structured interviews with 8 directors and staff from 4 ECE services

Theme	Supporting quote
**Enablers**	
1. Government funded breakfast program	(R1) *I think with the breakfast program in kinder, it's taken away the stigma. There's a number of families that have food security issues and actually can't provide a healthy lunch box or enough food for their children to eat. So, if they know that the children are going to be provided with sandwiches and yogurt and fruit in the morning, then it doesn't matter so much if they don't have healthy options for breakfast. So that's been a positive thing for our families that are vulnerable that just don't have the money to feed their children adequately*
	(R2) *We operate two ECEC centres within the same building: one for Kinder and the other for LDC. We successfully applied for government funding for breakfast provision at Kinder, but unfortunately, there was no opportunity to do the same for the LDC………* *While children in Kinder have nutritious and delicious government-funded breakfast provided, children in LDC were all looking at them and smelling the yummy breakfast, and kept asking us to be able to have that breakfast, instead of what they had, such as bread and milk. It was a challenge for us to ensure a nutritious breakfast for children. Sometimes, we've seen also some families with financial struggles to provide a healthy breakfast at home to children, but here in LDCs, we don't have any options to provide healthy breakfast options like Kinder*
2. Nutrition Training and Knowledge	*(M1) When I first started, it was Nutrition Australia. I'm not a hundred percent now, I think they've changed their name, but I always go back to those sorts of guidelines because it contains your five groups, how many portions they need per day, and how many grams within those portions. So that's my big thing to go to. This is what they need for their day, and this is how we can provide it for them* *(M2) When we design our menus, we make sure that they're getting their nutritional value that they require during the day from here to start with this, lower the sugary foods and up with the fibre and the protein and everything*
**Barriers**	
3. Time constraints	*(M3) Breakfast can start from 7.00 am but sometimes some kids come a little early, so depending on the arrival* *(R3) If children come after 8:15am, that's when breakfast ideally closes. But sometimes if parents come around 8:20am and request us, that's fine. However, at that time only toast is available. No cereals* *(M4) We have cereals, toast, milk and spreads such as vegemite and margarine. That’s all about it. The reason why we have a simple breakfast option is because we need to make it easier. Also, children like toast*
4. Budget limits	*(R1) Finance. Because it's sticking to the budget. That's the really hard part. And you know, we're a very big centre here ensuring that we meet all of their daily needs throughout the whole day, plus breakfast, plus, morning tea, lunch, afternoon tea, late snack, all the other meals that we provide. Budget is a big constraint that makes it harder sometimes to have everything we need* *(R2) Because like you look at watermelon, it's like some 30 Australian dollars a kilo. So obviously that's not even an option. Strawberries also aren't an option. So having more money would be a larger variety of fruits. And we'd be able to have a lot more cheese and stuff like that, that children love or even like sliced meats or something that you can have with it. But budget doesn't allow for that sort of expansive variety.”* *(R4) Barrier could be sometimes if a delivery is not on time. Especially for bread. Because for cereals, we always have in our pantry. But, uh, bread and milk, if a delivery is not on time. But that also, we, we try and prevent it by ensuring the delivery comes before Monday morning*
5. Inadequate staffing for individual child supervision	*(M1) I think sitting with them and enjoying their breakfast, you know, just it's like a meeting place. So, they come, and they discuss food or what they did last night. So, it's a communal type thing* *(M3) We often do all the breakfast in one room. About eight o'clock we have about 22 kids in one room for breakfast. At that time, we have to separate them* *(R3) On average, we have 20 to 22 children eating breakfast together regardless of the age of children and classroom of them* *(M4) In the morning when the staff comes to the centre, they ensure that the breakfast trolley is ready. That trolley should also have enough plates, enough bowls, enough spoons, tongs to serve. And chopping board should be ready for the toast. Educators cut the toast into pieces for them, just to make it easier for them to have a shorter piece. So, we cut one bread into six pieces. To effectively manage all the tasks associated with the morning routine, there is a need for additional staff who are well-versed in the specific responsibilities during this time* *(R4) We don't allow casual educators at breakfast time because of the allergies and stuff. They're not fully aware. So, we should remember all the details of children to save them*
6. Limited awareness of nutrition guidelines or access of nutrition training	(R1) *I don't know how healthy vegemite is and how amount I can provide this to children if children keep wanting. It's been salty so I think where we stop* (R2) *The most times that are probably not the best is when the children actually come in with the McDonald hash brown in their hand, or, you know, the, the packet of salt and vinegar chips that they're having for breakfast. That can be a little bit challenging to try. We generally just speak to the parent. We don't take the food off the children, but we'll generally just ask the child to sit at the at the table and finish their breakfast before they go and play* *And then we'll speak to the parents about appropriate food choices for children. But it is tricky to have those conversations because like I said, a lot of these families have food security issues. And if the only thing they've got in the cupboard is a packet of chips or a sweet biscuit, well that's what they'll give their child as opposed to not giving them anything at all* (M3) *Sometimes it takes a little while for the children to get used to having a breakfast, if they're not used to having it at home or a, a healthier type of breakfast, they might be using* *It's hard. But some of them are used to having a sweet biscuit or McDonald's or a flavored milk. That's their breakfast. So, it's disappointing that that's the case, but that's not their fault. It's not their parents' fault, it's how they were raised but that gives us the challenge. Now we have to educate them about what's a better option, not a wrong option, just a better option. So yeah, that's probably the most challenging*

#### Enabler 1: Government funded breakfast provision

One interviewee highlighted government-funded breakfast provision as a crucial enabler in these ECEC settings, ensuring that children received nutritious breakfasts, including options such as sandwiches and a variety of vegetables, yogurt, and fruit, all in accordance with the guidelines (R1). This interviewee also noted that this initiative could reduce stigma related to food insecurity in ECEC centres, leading to a significant improvement in the overall well-being and the assurance of a healthy breakfast for the children. Two interviewees (R1, R2), both working in Regional ECEC services, described families at their ECEC services struggling to provide healthy foods to their children. These two interviewees emphasised that government support in providing a healthy breakfast could significantly help these children and families (R1, R2). However, a clear challenge existed. This funding appeared to be primarily directed at Kindy attendees (aged 3–5years), unintentionally overlooking a significant portion of children under the age of 3 years attending LDCs who could equally benefit. One interviewee emphasised that it was challenging for LDCs to ensure a nutritious breakfast for their children (R2).

*“I think with the government funded breakfast provision in kindy, it's taken away the stigma. There's a number of families that have food security issues and actually can't provide a healthy lunch box or enough food for their children to eat. So, if they know that the children are going to be provided with sandwiches and yogurt and fruit in the morning, then it doesn't matter so much if they don't have healthy options for breakfast at home. So that's been a positive thing for our families that are vulnerable that just don't have the money to feed their children adequately (R1)”*

*“We operate two ECEC centres within the same building: one for Kindy and the other for LDC. We successfully applied for government funding for breakfast provision at Kindy, but unfortunately, there was no opportunity to do the same for the LDC………While children in Kindy have nutritious and delicious government-funded breakfast provided, children in LDC were all looking at them and smelling the yummy breakfast, and kept asking us to be able to have that breakfast, instead of what they had, such as bread and milk. It is such a shame and a challenge for us to ensure a nutritious breakfast for children at LDC. Sometimes, we've seen also some families with financial struggles to provide a healthy breakfast at home to children, but here in LDC, we don't have any options to provide healthy breakfast options like Kindy (R2).”*

#### Enabler 2: The use of nutritional guidelines

Interviews highlighted the significant role of nutrition guidelines in shaping the breakfast practices within ECEC settings. One interviewee emphasised the Nutrition Australia guidelines as a primary source of nutrition knowledge (M1). By utilising this knowledge, this interviewee aimed to provide well-balanced and nutritious meals to meet the children's dietary needs (M1). Additionally, one regional staff member shared that they had created their own tailored recipes based on these guidelines (R2).

*“When I first started, it was Nutrition Australia……. I always go back to those sorts of guidelines because it contains your five groups, how many portions they need per day, and how many grams within those portions. So that's my big thing to go to. This is what they need for their day, and this is how we can provide it for them (M1).”*

*“It [nutrition guideline book] helped me a lot in creating healthy menus for the kids. I even made my own recipe book using it, which has been incredibly useful in my daily work! (R2)”*

#### Barrier 1: Time constraints

The breakfast service showed similarity in start time, with breakfast potentially beginning from 7 am, although some children arrived a little earlier, leading to flexibility in the actual start time based on their arrival (M3). However, this flexibility was limited by the need to provide breakfast before the start of daily curriculum activities. The desired closing time for breakfast was approximately 8:15 am (R3). Nevertheless, there were instances when parents requested a slightly later breakfast service. In such cases, only toast was offered, and cereals were not available at that time (R3). This variability in breakfast service utilisation is intentionally designed to accommodate the diverse needs and schedules of the children and their families, ensuring that breakfast is available to as many children as possible. However, because of these time constraints, the selection of breakfast items becomes prioritised for convenience and ease of preparation.

“*Breakfast can start from 7.00 am but sometimes some kids come a little early, so depending on the arrival (M3)”*

*“If children come after 8:15am, that's when breakfast ideally closes. But sometimes if parents come around 8:20am and request us, that's fine. However, at that time only toast is available. No cereals (R3)”*

Regarding specific breakfast food items, a few interviewees indicated that the menu was designed to be easy and quickly prepared, with a focus on using readily available ingredients (R3, M4). Additionally, one interviewee emphasized that the aim was to provide convenient and practical options during the busy morning time at ECEC centres without compromising on the quality and nutritional value of the breakfast served (M4).

*“We have cereals, toast, milk and spreads such as vegemite and margarine. That’s all about it. The reason why we have a simple breakfast option is because we need to make it easier. Also, children like toast (M4)”*

#### Barrier 2: Budget limits

Budgets are crucial for ensuring that breakfast provision includes a diverse menu and maintains nutritional balance. Several interviewees underscored the considerable challenge posed by budget constraints when it comes to providing a healthy breakfast (R1, R2). One interviewee highlighted that adhering to a budget was particularly difficult for the large ECEC centre, as it had to prepare all the children's daily needs, including breakfast, morning tea (snack), lunch, afternoon tea (snack), and late snacks, with limited financial resources (R1).

Furthermore, services reported that these budget constraints for food provision directly influenced the availability of various food items, especially during breakfast, which is considered optional for children. One of the interviewees mentioned that fruits like watermelon and strawberries were not feasible options for the breakfast menus due to their expense (R2). As a result, the ECEC centre's ability to provide a diverse range of items that children typically enjoy was constrained.

“*Finance. Because it's sticking to the budget. That's the really hard part. And you know, we're a very big center here ensuring that we meet all of their daily needs throughout the whole day, plus breakfast, plus, morning tea, lunch, afternoon tea, late snack, all the other meals that we provide. Budget is a big constraint that makes it harder sometimes to have everything we need. (R1)”*

*“Because when you look at watermelon, it's like some 30 Australian dollars a kilo. So obviously that's not even an option. Strawberries also aren't an option. So having more money would be a larger variety of fruits. And we'd be able to have a lot more cheese and stuff like that, that children love or even like sliced meats or something that you can have with it. But budget doesn't allow for that sort of expansive variety. (R2)”*

#### Barrier 3: Inadequate staffing for individual child supervision

Regarding breakfast environment, educators sat with the children, sharing breakfast together, and engaging in conversations about food or their activities, fostering a sense of connection and trust before the busy morning. One interviewee emphasised that breakfast time provided the opportunity to promote individualised attention and support, creating a welcoming and communal atmosphere (M1).

*“I think sitting with them and enjoying their breakfast, you know, just it's like a meeting place. So, they come, and they discuss food or what they did last night. So, it's a communal type thing* (*M1).”*

The morning routine at the centre typically involves gathering approximately 20–22 children together in one room between 8 am and 8:30 am, irrespective of age. After breakfast children are separated into their respective classrooms by age (M3, R3).

“*We often do all the breakfast in one room. About eight o'clock we have about 22 kids in one room for breakfast. At that time, we have to separate them (M3).”*

*“On average, we have 20 to 22 children eating breakfast together regardless of the age of children and classroom of them (R3).”*

Staff highlighted a need for additional staff to help with management until children finished breakfast and returned to their respective rooms. One interviewee mentioned taking the initiative to cut the toast into smaller pieces, making it easier for the children to handle, while others attend to the children during breakfast (M4). Furthermore, it was emphasized by another interviewee that only full-time educators or breakfast experienced staff were allowed to serve the foods and supervise the children during breakfast time due to concerns about allergies and the need to have full awareness of each child's details (R4).

*“In the morning when the staff comes to the centre, they ensure that the breakfast trolley is ready. That trolley should also have enough plates, enough bowls, enough spoons, tongs to serve. And chopping board should be ready for the toast. Educators cut the toast into pieces for them, just to make it easier for them to have a shorter piece. So, we cut one bread into six pieces. To effectively manage all the tasks associated with the morning routine, there is a need for additional staff who are well-versed in the specific responsibilities during this time (M4).”*

#### Barrier 4: A lack of confidence in interpretation and application of nutrition guidelines

One interviewee expressed uncertainty about whether Vegemite is healthy and the appropriate serving amount for children based on the guidelines when children continuously request it (R1). Another interviewee mentioned challenges arising from a lack of interpretation of the nutritional guidelines when children brought unhealthy breakfast items like packaged hash browns or chips (R2). While the interviewees wanted to teach children about healthier breakfast options, their confidence of interpretation or practical application of nutritional guidelines seemed limited.

*“I’m not confident about how much to provide when children continue to request Vegemite and I’m not sure if I’m following the guidelines (R1).”*

## Discussion

The current study provides unique insights into on-site breakfast provision at Victorian ECEC centres, Australia, focusing on the types of breakfast foods offered as well as the factors that enable or are barriers to the provision of healthy breakfasts on-site. Breakfast cereals, bread, and milk were the most commonly provided items, while fruits and vegetables were less frequently offered in ECEC settings. Of the 50 ECEC centres surveyed, over 80% provided low to medium quality breakfasts, based on the two HEAS guidelines [15, 27], with many of these centres located in socio-economically disadvantaged areas. To improve the quality of breakfast provision, our qualitative findings explored government-funded breakfast programs and nutritional guidelines as key enablers, while a lack of time, staff, budgets, and confidence in using the guidelines were identified as significant barriers.

In Australia, the Victorian Healthy Eating Advisory Service (HEAS) provided two key frameworks to improve nutritional quality in childhood: the Healthy Choices Food and Drink Classification Guide [27] and the Breakfast Menu Recommendations for ECEC Services [15]. The guidelines classified foods using a traffic light system—GREEN (best choices), AMBER (choose carefully), and RED (limit) [27], and encouraged the inclusion of whole grains, reduced-fat dairy, fruits, vegetables, and protein-rich foods at breakfast [15]. Despite these recommendations, our findings revealed that 80% of centres (n = 50) provided low to medium-quality breakfasts, primarily consisting of cereal, bread, and milk, with limited inclusion of fruits and vegetables. Similarly, Park et al., have reported that Australian children up to age 5 years frequently consumed breakfast cereals, bread, and milk, while vegetables and fruits were seldom included [29]. These findings indicated the need to strengthen the implementation of breakfast guidelines to improve the nutritional quality of breakfasts provided to children.

In many Western countries, breakfast typically includes ready-to-eat cereals, which are considered a convenient and popular option. However, this study identified significant variations in the nutritional quality of breakfast cereals provided in ECEC settings, indicating that not all cereals offered are equally nutritious. Our online survey findings showed that Weet-Bix ^™^, Nutri-Grain ^™^ and Rice Bubbles ^™^ were commonly provided in ECEC settings. According to HEAS guidelines [15, 27], Weet-Bix ^™^ contained significant levels of fibre and whole grains, while Nutri-Grain ^™^ and Rice Bubbles ^™^ had higher levels of added sugars and lower fibre content [15, 27]. It is worth noting that the type of cereal significantly influenced the overall nutritional value of breakfast and the quality of breakfast provision. To enhance breakfast quality, ECEC centres should prioritise offering a variety of low-sugar, wholegrain cereals including Weet-Bix TM, oatmeal, whole grain flakes, or untoasted muesli, providing children with diverse nutrients and flavours.

Our findings also revealed that a majority of ECEC centres provided milk as part of their breakfast offerings. Dairy products, including milk, play a crucial role in providing essential nutrients such as calcium and vitamin D (if fortified) for young children [30]. From our findings, over 90% of ECEC centres in our samples (n = 50) predominantly offered full-fat milk. Dietary recommendations suggest a gradual transition to consuming lower-fat milk options for children over the age of 2 years [31, 32], reducing their intake of energy from saturated fat (no more than 10%), including fat in milk [31, 33]. Similar to our findings, full-fat milk was also predominantly served in ECEC centres in Mexico [34]. However, 1% reduced-fat milk was the primary choice offered in ECEC centres in the US [35]. Considering the typical age group of children in ECEC centres (usually 2 to 5 years old), current breakfast menu planning guidelines [15] could benefit from updates to incorporate reduced-fat or low-fat milk as part of breakfast provisions, ensuring alignment with national dietary recommendations.

Previous UK research found that offering vegetables at breakfast for children is uncommon in Western countries, with practical challenges identified as one of the key barriers to providing vegetables during this meal [36, 37]. Consistent with findings from the UK [36, 37], our study also found that vegetables were not commonly offered during breakfast within Australian ECEC centres despite being recommended within the HEAS Breakfast Menu Recommendations for ECEC Services [15]. Including vegetables in the breakfast menu can be a challenge at ECEC centres, considering that it may not align with the traditional cultural norms in many Westernised countries, including Australia [29, 36]. Given that children’s vegetable consumption falls well short of dietary recommendations, ECEC centres provide an important opportunity to expand vegetable offerings during breakfast, as many Australian children begin their day at ECEC centres [36]. The specific types of vegetables and their preparation methods may depend on the age of the children, dietary preferences, and cultural considerations. Collaborative efforts among ECEC centres, nutrition professionals, and policymakers are important to establish comprehensive guidelines and recommendations that prioritise the provision of vegetables during breakfast at ECEC centres. This involves addressing cultural challenges and exploring creative ways to do so, such as recent trends to include avocado, tomato, and baked beans [28].

To improve the quality of breakfast provision in ECEC settings, our qualitative findings identified the Australian government-funded breakfast provision for ECEC services [38] as an example of enabling high quality breakfast provision in early childhood setting. However, the current opportunity was exclusively available to kindergartens (Kindy), with the exclusion of LDCs, thereby limiting the provision to all children attending the ECEC service. It is worth considering the expansion of this government-funded breakfast provision to include LDCs to ensure equal opportunity for all children to access a nutritious breakfast. It will be important also to further explore potential administrative barriers that may be reducing uptake of this service. Additionally, our findings indicated that this concern was raised only by staff working in regional centres. Further investigation is needed to determine whether similar issues are also occurring in metropolitan centres. Therefore, it is essential to establish a system that ensures equitable distribution of funding across regions and centre types, promoting fair and consistent access to resources for all ECEC services.

Additionally, our study revealed that the awareness and utilisation of nutrition guidelines among ECEC staff played as a facilitator in providing healthy breakfast options within ECEC centres. Similar to breakfast provision, the effective utilisation of nutrition knowledge-based guidelines was emphasised in improving the overall quality and nutritional value of main meals (e.g., lunch, morning/afternoon snacks) at ECEC centres [20]. Staff members who were knowledgeable about nutrition guidelines could provide evidence-based knowledge and skills necessary to plan and offer nutritious main meal options.

In contrast, we also identified that a lack of confidence in interpretation and application of nutrition guidelines among ECEC staff was a potential barrier to providing healthy breakfast options. Similarly, a US study [39] examined the lack of confidence in the interpretation of nutritional guidelines among American ECEC educators when providing food to children on-site at ECEC centres. Given the likely importance of proper nutrition guideline utilisation among ECEC staff in providing healthy breakfasts at ECEC centres, it is recommended to enhance staff confidence in practical application of current nutrition guidelines and provide support for healthy breakfast provision through nutritional education. This will equip educators with the necessary knowledge and skills to align breakfast menus with nutritional guidelines effectively, ultimately promoting healthier breakfast options for children attending ECEC centres. In addition to nutrition guidelines, guidelines on mealtime practices for educators might be considered. Such guidelines have been already utilised in Sweden [40] and the United Kingdom [37] and have contributed to enhancing the breakfast environment.

Furthermore, our qualitative findings highlighted that limited food budget was a barrier to healthy breakfast provision within ECEC centres. ECEC staff expressed concerns about the financial challenges of procuring diverse and nutritious breakfast options. A study conducted by Gabor et al. revealed that budget constraints often restricted ECEC centre ability to offer a wider range of wholesome and high-quality food items [41]. In our study, we identified that ECEC centres with higher socioeconomic position (SEP) (based on the Socio-Economic Indexes for Areas (SEIFA) [42]) were more likely to provide higher-quality breakfast. It is possible that ECEC centres in higher SEP areas may have access to larger food budgets and other staff related capacities (e.g. professional development) [43, 44]. As breakfast provision is considered optional in guidelines [15], it is possible that centres in low SEP areas may not prioritise offering a diverse range of breakfast options due to budget constraints.

To address budget limitations, Australian ECEC centres might adopt strategies used in the USA's ECEC centres participating in Child and Adult Care Food Program (CACFP) [45]. These strategies include cost-effective procurement methods like purchasing qualifying produce from food service providers, local grocery stores, or warehouse stores through partnerships. Research in the USA [46] has shown that over half of ECEC centres in their study used these approaches, making it easier for them to purchase nutritious foods for children within their budget. Additionally, some ECEC centres in the USA collaborated with community organisations to access affordable food and seek partnerships or government funding dedicated to improving food provision, helping them overcome financial constraints and ensure children receive nutritious on-site food provision [46].

Moreover, the limited availability of experienced or full-time staff who were familiar with the children in attendance was identified as one of the barriers in our study. This constraint is particularly significant due to the diverse ages and dietary needs of those sharing the breakfast space during this time. It was highlighted that part-time or casual staff may not be familiar with the individual characteristics of the children, especially when multiple groups of children gather together during breakfast. Skilled full-time teachers overseeing breakfast ensure quick, balanced, and customised breakfast options tailored to each child, all while effectively managing their time. In this context, our study emphasised the necessity for experienced full-time staff during breakfast hours to cultivate a more favourable breakfast environment.

A strength of this study is its multi-method approach, using a combination of quantitative and qualitative research methods. Importantly, it is the first study to explore on-site breakfast provision at ECEC centres using this comprehensive approach. This provided a deeper description of breakfast provision quality and associated factors in ECEC centres. The quantitative component provided us with empirical data to examine the association between SEP and breakfast quality, enhancing the robustness of our findings. The qualitative component provided valuable insights into the perspectives and experiences of ECEC centre staff regarding barriers and enablers to providing nutritious breakfasts.

A limitation of our study is that we did not capture actual food consumption data during breakfast. This was beyond the scope of our research objectives, particularly given the challenges as Victoria emerged from the Covid epidemic. Such data would enable quantitative evaluation of actual breakfast consumption and quality in reference to total daily energy and nutrient intakes of children in ECEC settings. We recommend collecting the amount of food consumed during breakfast to comprehensively evaluate breakfast quality in future research. Although reflexivity was integrated throughout the research process to reduce potential biases, we acknowledge that our assumptions and interactions with participants may have impacted the data collection and analysis. Additionally, it is important to note that our results, both qualitative and quantitative, may not be fully representative of all ECEC centres and thus may not be generalisable within the Australian context and beyond. Future research with a larger and more diverse sample is recommended.

## Conclusion

This study provides important insights into the quality of breakfast provision in ECEC centres. Study findings indicate that most ECEC centres (over 80%) offer mid- to low-quality breakfast options, with minimal provision of vegetables and fruits during breakfast. Additionally, the study highlights an association between higher SEP and higher-quality breakfast provision, revealing socioeconomic disparities in provision of nutritious breakfasts. Enhancing practical application about nutrition guidelines and providing targeted support (such as a government funded breakfast provision and full time staff) may have the potential to mitigate barriers such as time/staff/budget constraints and limited confidence in utilisation of nutrition guidelines for healthy breakfast provision. To further improve breakfast provision in ECEC settings, simplified breakfast provision guidelines—including visual tools, menu templates, and quick-check lists—should be developed to help ECEC staff efficiently prepare and provide nutritious breakfasts. Additionally, relevant training for ECEC staff could focus on breakfast menu planning, portion sizes, age-appropriate food options, and quick breakfast recipes to support practical implementation within busy morning routines. Further studies are needed to identify viable and actionable solutions for implementing these improvements.

## Supplementary Information

Below is the link to the electronic supplementary material.

Supplementary file1 (DOCX 402 KB)
