# Supplementary material for: On-site breakfast provision in early childhood education and care (ECEC) services in Australia: a multi-method investigation
Source: Eur J Nutr. 2025 Feb 1;64(2):78. doi: 10.1007/s00394-025-03590-4 (PMC11787215; doi:10.1007/s00394-025-03590-4)
Supplement: Supplementary file 1 — Supplementary file1 (DOCX 402 KB) [file 394_2025_3590_MOESM1_ESM.docx]

**Supplementary document 1 – Online Survey questionnaires**

Deakin Long Day Care BREKKIE Study

Thank you for your interest in participating in this study.

**Centres are eligible for this study if they:**

1. **Are located in Regional Victoria AND**
2. **Operate for at least 8 hours per day AND**
3. **Provide breakfast on-site**

More information about the study is available in the attached Plain Language Statement which you can download and keep if you like. If you have any questions please email the research team at [ldcstudy@deakin.edu.au.](mailto:ldcstudy@deakin.edu.au)

[Attachment: "LDC Surveys PLS and consent.pdf"]

Name of Long Day Care Centre

Postcode of Long Day Care Centre

Please indicate your ability to participate in this Our Centre is not eligible for this study

study by selecting the relevant box below: Our Centre is eligible for this study but we would prefer not to participate


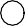

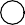


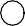
 Our Centre is eligible for this study and we would like to participate

Thank you for your time.

I give consent to participate in this 10-minute survey:

Yes No


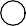

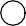


By selecting YES I am confirming that:

I have read and understand the attached Plain Language Statement. I freely agree to participate according to the conditions in the Plain Language Statement. I have downloaded a copy of the Plain Language Statement and Consent Form to keep. I understand that the researchers have agreed not to reveal my identity and personal details, including where information about this project is published, or presented in any public form.

Thank you for agreeing to participate in this study.

*Everyone who completes this survey will receive a **$20 Coles/Myer gift card**

Your First Name

Your Last Name

Your email address

What is your country of birth?

Do you speak a language other than English? Yes, please specify: No

**Section A: About your centre and role**

A1. How long have you worked in your current role as Less than 12 months Director? 1-2 years


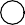

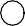

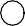

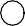

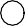


2-3 years

3-4 years

Over 4 years

A2. Please select your highest level of educational University Degree

attainment Trade, Apprenticeship, Diploma, certificate


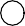

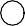

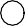

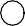


Year 12

Year 11 or below


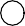

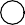


A3. Have you had any training in nutrition to support Yes, please list these: you in your role? No

A4. Is your centre privately managed or community Private

owned? Community


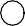

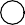


A5. Total number of staff at your long day care centre (including the director)

A6. Total number of enrolments (aged 1-5 years) at your centre

A7. Please indicate which of the following age groups you have at your centre. Select all that apply.

< 1 year

1. year
2. years
3. years
4. years
5. years

(Mark all that apply)

A8. Please describe whether your meal service is Progressive meal


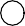

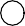

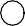


progressive, standard or flexible. Standard meal Flexible meal

Progressive meal = an extended time for the meal where the food is made available and children may opt to

have some food when they like and leave when they are finished.

Standard meal = a set meal time frame where children are asked to leave activities and come together for

the meal.

Flexible meal = a meal that is provided when the children are hungry.

**Section B: Breakfast provision in your centre**

B1. Which of the following most closely describes how food is usually served to children during BREAKFAST (select only ONE)?

Children serve themselves most foods and decide how much to take


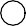

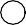

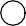

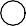


Children serve themselves most foods and staff decide how much the child can take Staff serve most foods and the child decides how much to take

Staff serve most foods and decide how much to give to each child

B2. What time does breakfast usually start?

B3. What time does breakfast usually finish?

B4. On average how many children eat breakfast per day for the following age groups:

< 1 year: 1 year:

1. years:
2. years:
3. years:
4. years:

B5. How many staff are usually involved in breakfast provision?

B6. Does cook/chef usually work during breakfast time? Yes

No


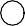

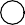


**B7. Who is mainly responsible for the following regarding breakfast provision? (Mark all that**

**apply)**

a designated cook educators directors other

Breakfast food budgeting Breakfast menu planning Breakfast food preparation

Supervising children during breakfast

B7.1. If you indicated "other" in the previous question, please specify here

**B8. Do any of these staff have training in nutrition that includes understanding of dietary**

**guidelines? (note: in this case dietary guidelines are referring to child-specific guidelines on food groups and amounts needed per day)**

Yes No Unsure

Staff mainly responsible for
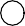

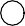

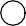
 breakfast food budgeting

Staff mainly responsible for
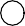

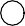

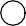
 breakfast menu planning

Staff mainly responsible for
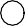

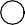

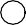
 breakfast food preparation

Staff mainly responsible for
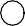

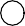

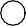
 supervising during breakfast

**B9. Please tell us whether you usually provide the following at breakfast:**

Breakfast cereal Bread

Porridge Milk Cheese Yoghurt Fruit Vegetables

Never Sometimes Most of the time Always


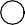

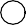

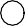

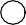

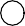

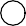

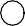

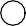

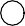

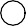

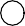

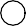

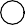

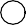

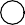

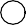

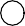

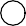

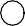

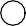

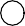

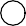

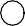

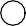

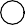

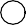

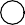

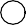


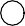

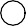

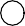

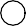


B10. Mark all BEVERAGES served to most children at breakfast (Mark all that apply)

100% fruit juice other fruit juice sweet tea water

other drinks - please specify: milk

plant-based milk (eg: soya, oat, rice, almond) not served

B11. Mark all BREADS served to most children at breakfast (Mark all that apply)

Bread/ toast Wrap/ flatbread Bagel

Muffin Pancakes French toast

Other - please specify: Not served

B11.1. What type of bread/toast was served? (Mark all that apply)

white wholegrain/wholemeal fruit

rye

other - please specify:

B12. What type of CEREALS were mostly served at breakfast?

cold cereal porridge (eg: oats)

other - please specify not served

B12.1. Mark all CEREALS served to most children at breakfast (Mark all that apply)

Weet-bix Coco Pops Nutri-grain Muesli Milo

Rice Bubbles Cornflakes Sultana Bran

Other - please specify:

B13. Mark all OTHER BREADS and GRAINS served to most children at breakfast (Mark all that apply)

breakfast bar (e.g. granola/ muesli, cereal bar) - brand name: biscuits (e.g. chocolate chip)

crackers rice cakes

other - please specify: not served

B14. Mark all dairy products served to most children at breakfast, then specify characteristics

Milk Cheese Yoghurt

Other dairy products Not served

B15. Mark if FRUIT is served to most children at breakfast. Do not include juice.

Fresh - type (e.g: banana) Frozen - type (e.g: berries)

Canned in juice (100%) - type (e.g: peaches) Canned in syrup - type (e.g: peaches)

Dried - type (e.g: raisins)

Other - please specify (e.g: mashed banana): Not served

B16. Mark if VEGETABLES are served to most children at breakfast. Do not include juice.

Fresh - type (e.g: tomato, avocado) Cooked - type (e.g: mushroom) Other - please specify:

Not served

B17. Mark any CONDIMENTS served to children at breakfast (Mark all that apply)

oil, butter, or margarine cheese

honey jam

peanut butter sugar / brown sugar syrup

other (eg: vegemite) - please specify: Not served

B18. Are any other food items served at BREAKFAST? No

(eg: scrambled eggs) Yes - specify what these other food items are:


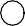

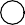


B19. Please describe how your BREAKFAST menu is developed? For example - do you do this by yourself,

does someone assist you, is it provided to you from an external source.

**B20. Please attach electronic copies of your centre menu, showing your BREAKFAST menu**

**choices, using the "upload file" links below. Where possible please also upload recipes used for any cooked breakfast items.**

B20.1. Additional upload (if required)

B20.2. Additional upload (if required)

**Thank you for completing this survey.**

To receive your gift card please provide us with your postal address.

Please note: Your postal address will not be used for any other purpose and will not be shared.

Would you also like to participate in a 1-day ONSITE VISIT and follow-up INTERVIEW?

This onsite visit will be conducted by one of our researchers to observe breakfast provision. Follow-up interviews with centre directors and/or staff responsible for breakfast provision will explore challenges when providing breakfast and developing healthy breakfast menus within childcare centres. Interviews will be voluntary, take about 30-40minutes, and be scheduled at a time convenient to you in-person, by telephone or via Zoom. Centres will receive a $50 gift card voucher and interviewees will receive a $20 gift card voucher for their time.

More information about the site visit is available in the attached Plain Language Statement.

If you have any questions please email the research team at [ldcstudy@deakin.edu.au.](mailto:ldcstudy@deakin.edu.au)

[Attachment: "LDC Surveys PLS and consent.pdf"]


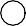
 Yes
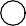
 No

Would you also like to receive a copy of the study findings?
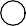
 Yes
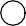
 No

Thank you so much for your time. Please press SUBMIT

**Supplementary document 2 – Observational study audit tool**

*Page 1*

EPAO: ECEC Nutrition Environmental Observation – BREAKFAST AUDIT TOOL

The aim of this survey is to evaluate ECEC settings to measure nutrition environmental characteristics.

The following survey is to be completed by a member of the research team.

In each section, please describe what happened TODAY.

Date of Observation

Centre Name

Survey Completed By: (Your Name)

Number and ages of children being observed for this data form (Mark all that apply)

- 1 year – number ___________________
- 2 years – number ___________________
- 3 years – number ___________________
- 4 years – number ___________________
- 5 years – number ___________________

**Did you observe any of the following after breakfast?**

The educator led a planned nutrition lesson with the children
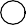
 Yes
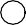
 No

The educator led a healthy eating activity
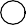
 Yes
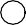
 No

The educator led a cooking activity
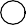
 Yes
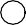
 No

The educator read a book to the children that included positive messages about

healthy eating
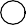
 Yes
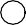
 No

The educator offered food to calm an upset child
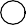
 Yes
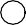
 No

(What was served: ________________ )

**BREAKFAST**

**What time did breakfast start? ____________ finish? ___________________**

**How many staff were engaged in supporting breakfast? _________________________________**

**Who cooked/prepared breakfast? (cook, educator, other) _________________________________**

**Who served breakfast? (cook, educator, other) _________________________________**

**Which statement best describes food preparation for BREAKFAST?**

- Prepared in kitchen and served in the classroom
- Prepared and served in the classroom
- Other: ______________________________________________________

**Describe this mealtime**

- Progressive
- Standard
- Flexible

*Progressive* = an extended time for the meal where the food is made available and children may opt to have some food when they like and leave when they are finished

*Standard* = a set meal time frame where children are asked to leave activities and come together for the meal

*Flexible* = a meal that is provided when the children are hungry

**The mealtime was organised in a way that was relaxed and enjoyable**


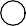
 Yes
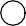
 No

**Staffing arrangements supported a relaxed and enjoyable mealtime**


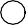
 Yes
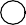
 No

**Children played a role in setting up mealtime and cleaning up afterwards**


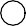
 Yes
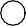
 No

**Educators supported children to collaborate, learn from and help each other**


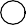
 Yes
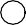
 No

**Was the TV on during breakfast?**


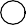
 Yes
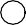
 No

**Was child size appropriate tableware used (eg: smaller plates and cups)?**


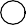
 Yes
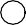
 No

**A variety of healthy imagery (posters, books, etc) is visible to the children?**
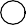
 Yes No

**A variety of unhealthy imagery (posters, books, etc) is visible to the children?**

Yes No

**Which of the following practices most closely describes how food was served during breakfast?**

**(Mark only one)**

Children serve themselves and decide what foods and portion sizes to take

Children serve themselves but staff decide what foods and portion sizes to take

Staff serve most foods and children decide what portions they want

Staff serve most foods and decide what portion size to give to children

Food arrives already portioned on each child's plate

Children bring food from home

**During breakfast, did the educator consume any of the following in front of the children?**

**(Mark all that apply)**

fast food

a sweet snack (donuts, pastries, cookies, lollies)

fruits or vegetables

sugar sweetened beverage

the same foods as the children

**Did the following interactions occur between the educator and the children during breakfast?**

The educator encouraged children to sit around the table during the mealtime

Yes No

The educator took a moment to settle the children before eating

Yes No

The educator talked with the children about the foods they were eating

Yes No

The educator talked with the children while they were eating (about anything)

Yes No

The educator role modeled eating healthy foods

Yes No

The educator encouraged (not forced/coerced) children to try foods on their plates

Yes No

The educator praised a child for eating

Yes No

The educator praised a child for eating healthy foods

Yes No

The educator sat with the children during the mealtime

Yes No

The educator pressured a child to eat

Yes No

The educator served second helpings only after confirming children were still hungry

Yes No

The educator served second helpings without confirming children were still hungry

Yes No

The educator provided alternate food if the child did not like what was being served

Yes No

If a child ate less than half their meal/snack, the educator removed the child’s plate

only after asking if the child was full

Yes No

If a child ate less than half their meal/snack, the educator removed the child’s plate

without asking if the child was full

Yes No

The educator made children finish all the food on their plate before leaving the table

Yes No

The educator promised something other than food as a reward for eating

(“If you eat your fruit, we can play ball outside”)

Yes No

The educator used food as a reward or bribe for eating a less preferred food

(“You can’t have your biscuit until you eat your apple”)

Yes No

The educator used food as a reward or withheld food as punishment for behaviour

(“If you clean up your toys, you can a second helping of food”)

Yes No

The educator was on their phone/computer during the mealtime

Yes No

The educator used an authoritative feeding style*

Yes No

**Definition: Authoritative feeding style - a balance between encouraging children to eat healthy foods and allowing children to make their own food choices. Providers use reason and education, rather than bribes or threats.*

**OBSERVATION NOTES:**

**BREAKFAST FOODS/BEVERAGES SERVED TO CHILDREN**

**Mark all BEVERAGES served to most children (Mark all that apply)**

- 100% fruit juice
- other fruit drinks
- sweet tea
- water
- other drinks – specify: ____________________
- milk - (Mark all that apply)
  - full fat
  - reduced fat
  - skim
  - flavoured milk (eg: vanilla, strawberry, chocolate)

**Mark all BREADS served to most children (Mark all that apply)**

- Bread / toast
- Wrap / flatbread
- Bagel
- Muffin
- Pancakes
- French toast
- Other – specify: __________________

**What type of bread products were served? (Mark all that apply)**

- white
- wholemeal
- multigrain
- cinnamon
- raisin/fruit
- rye
- unsure
- other – specify: _______________________

**Mark all CEREALS served to most children (Mark all that apply)**

****check labels of those marked – sugar>5g/serving; fibre >3g/serving)***

- Weet-bix
- Coco Pops
- Nutri-grain
- Muesli
- Milo
- Rice Bubbles
- Cornflakes
- Sultana Bran
- other – specify: _______________________

**Mark all OTHER BREADS and GRAINS served to most children (Mark all that apply)**

- breakfast bar (e.g. granola / muesli, cereal bar) [Brand name:_____________________]
- biscuits (e.g. chocolate chip)
- crackers
- rice cakes
- other – specify: _______________________

**Mark if FRUIT was served to most children. Do not include juice.**

- **Fruit was not served**
- **Fruit was served**
- Fresh – type eg: banana ____________________
- Frozen – type eg: berries ____________________
- Canned in juice (100%) – type: eg: peaches ____________________
- Canned in syrup – type eg: peaches ____________________
- other – specify: _______________________

**Mark if VEGETABLES were served to most children**

- **Vegetables were not served**
- **Vegetables were served**
- Fresh – type eg: tomato: ____________________
- Cooked – type eg: mushrooms __________________________
- other – specify: _______________________

**Mark any CONDIMENTS served to children (Mark all that apply)**

- oil, butter, or margarine
- cheese
- honey
- jam
- peanut butter
- sugar / brown sugar
- syrup
- other – specify: _______________________

**Were any other food items served at BREAKFAST today? (eg: scrambled eggs)**

No

Yes - specify what these other food items were:

**Supplementary document 3. Semi-structured interview guide**

Thank you for agreeing to participate in this interview.  As outlined in the Plain Language Statement I sent you, this interview is about understanding enablers or barriers to providing breakfast on-site at childcare. So, there are no right or wrong answers here, I’m just interested in your own views.

The interview will take 30 to 45 mins and will be audio-recorded as you confirmed before [If decline – take comprehensive hand notes].

**Before we start on the interview questions, I would like to reconfirm your details.**

- Can you please tell me what your role is within the centre?
- How long you’ve been working in this role?

1. Can you describe for me how breakfast is usually planned, prepared and served to children in your centre?

{prompts: any specific processes followed; what’s your role in planning, preparing, serving; how confident do you feel planning, preparing, serving breakfast to children and why}

1. How do you feel about being involved in planning/preparing/serving breakfast to children in your centre?

{prompts: uncertain, optimistic, confident, expect things to go well/go wrong}

1. When you hear people talking about “healthy breakfast for young children”, what does this mean to you?

{prompts: specific foods to eat, specific time to eat, will lead to benefits, etc)

1. What are your thoughts about childcare centres providing a healthy breakfast to children?

{prompts: do you think centres should provide breakfast? do you think it’s important for these breakfasts to be healthy?  Why / why not?}

1. Can you tell me about a good/pleasant experience you had providing breakfast to children in your centre. [open ended question]

1. I’d like to know more about what helps you to provide a healthy breakfast to children in your centre.

{prompts: any guidelines followed, any training done, job recognition, available time/budget…}

1. Can you tell me about an unpleasant experience you had providing breakfast to children in your centre. [open ended question]

1. I’d like to know more about any barriers that you think prevent you from providing a healthy breakfast to children in your centre.

{prompts: information/guidelines, training, time, budget, children’s preferences…}

1. What do you think is needed to help childcare centres provide a healthy breakfast?

[open ended question]

- Are there any other comments or anything you would like to add or change?
- Do you have any questions for me? [open response]

Thank you for participating in this interview.  Your participation will provide us with valuable insights on what supports are needed for healthy breakfast provision to children in childcare settings.
